# Comparing predictors of emotional intelligence among medical and nursing staff in national health system and military hospitals: A cross-sectional study in Greece

**DOI:** 10.3934/publichealth.2025034

**Published:** 2025-06-24

**Authors:** Vassiliki Diamantidou, Evangelos C. Fradelos, Athina Kalokairinou, Daphne Kaitelidou, Petros Galanis

**Affiliations:** 1 ICUs Department, 251 Air Force Military Hospital, Athens, Greece; 2 Laboratory of Clinical Nursing, Department of Nursing, University of Thessaly, Larissa, Greece; 3 Community Nursing Laboratory, Faculty of Nursing, National and Kapodistrian University of Athens, Athens, Greece; 4 Center for Health Services Management and Evaluation, Faculty of Nursing, National and Kapodistrian University of Athens, Athens, Greece; 5 Clinical Epidemiology Laboratory, Faculty of Nursing, National and Kapodistrian University of Athens, Athens, Greece

**Keywords:** emotional intelligence, healthcare professionals, national health system, military hospitals, Greece

## Abstract

**Background:**

Emotional intelligence (EI) is a crucial skill in the healthcare industry, closely related to empathy, communication, and stress tolerance. Although EI has been well researched among healthcare workers, there is little information comparing organizational structures.

**Objective:**

Our purpose of this study was to examine the EI of medical staff employed by the Armed Forces (military) and National Health System (NHS) hospitals in Athens, Greece, and investigate the connection between EI and professional or demographic traits.

**Methods:**

A cross-sectional study was conducted from November 2022 to July 2023 involving 1108 healthcare professionals (nurses and physicians) recruited through convenience sampling. Participants worked in ICUs, surgical, and medical departments of four military and three NHS hospitals in Attica EI was measured using the Greek-validated Wong and Law EI Scale (WLEIS). Bivariate and multivariable linear regression analyses were carried out. Analyses of bivariate and multivariable linear regression were performed.

**Results:**

Healthcare professionals in NHS hospitals demonstrated significantly higher EI scores across all dimensions compared to those in military hospitals (*p* < 0.001). Contrary to several earlier findings, male professionals showed higher values in emotional regulation and overall, EI. Higher EI is related to greater professional experience and permanent work status. Healthcare professionals who were working on rotated shifts reported higher scores for EI compared with those who were working morning shifts.

**Conclusions:**

The EI of medical and nursing staff is influenced by work experience, employment stability, and organizational structure. Military hospitals and the hierarchical structures of such hospitals may pose an obstacle to emotional growth and expression. This underscores the necessity of specialized EI training in these settings. Finally, these results highlight the necessity of the development of EI to enhance patient care and teamwork.

## 1. Introduction

Emotional intelligence (EI) refers to an individual's capacity to recognize, understand, manage, and influence their own and others' emotions [1]. Salovey and Mayer (1990) defined EI as the ability to understand, comprehend, and manage one's own and other people's emotions in a manner that can shape one's thoughts and actions. The key dimensions of EI include emotion perception, the ability to recognize emotions through expressions or sounds, use them to enhance thinking and creativity, understand the causes and consequences of emotions, and manage them to maintain mental balance and effectively respond to circumstances. EI, recognized as vital to personal and professional success, results from these components [2]. Goleman (1995) defines EI as the ability to recognize our own and others' emotions to self-motivate and effectively manage our emotions within intrapersonal relationships. Goleman's model of EI consists of five abilities: Self-awareness, which refers to the ability to understand our emotions and their consequences; self-regulation, which is the ability to control and adjust our emotions according to circumstances; motivation, which is the inner drive to achieve goals despite difficulties; empathy, which refers to the ability that ones have to understand and respond to the emotions of others; and social skills, such as the ability to establish and maintain positive interpersonal relationships [3]. Despite their similarities, these approaches exhibit multiple differences.

On the one hand Salovey & Mayer model emphasizes the cognitive and emotional processes that define EI, while the Goleman model focuses on the application of EI in domains such as leadership and interpersonal relationships, along with the skills necessary for the effective management of emotions across various contexts [2]. Furthermore, according to Salovey and Mayer, EI is a cognitive-emotional skill that includes perception, comprehension, control, and strategic use of emotions. The Wong and Law EI Scale, which operationalizes these skills, is used in this study with support from this framework. Goleman's model; on the other hand, it emphasizes the interpersonal and behavioral skills required for successful interaction in various contexts, such as the healthcare industry. These two models combined contribute a thorough framework for investigating how EI is influenced by components such as job position and organizational structure. A comprehensive examination of the differences in EI between healthcare workers in institutional contexts and professional levels is made possible by this integrated methodology.

Nowadays, EI has been identified as an important component for providing effective patient care in healthcare professions. There are several studies suggesting EI can be used to decrease anxiety and occupational stress and to enhance communication and collaboration between healthcare professionals and patients. In addition, since nurses are exposed to emotionally demanding circumstances, emotion management not only enhances patient care but also supports nurses' mental health [4]. Research reveals significant differences in EI between the medical and nursing staff, with nurses exhibiting higher levels of empathy, whereas physicians, due to their training, often demonstrate greater self-regulation of emotions. Thus, improving EI through appropriate educational programs can result in increased job satisfaction and effectiveness [5].

Moreover, EI is associated with optimal emotion management and the professional well-being of healthcare professionals. A study by Liu et al. (2023) among psychiatric nurses revealed that higher EI is associated with better identity, management, and regulation of their emotions. In addition, EI enables psychiatric nurses to respond to job stress and demands more effectively. Thus, training in EI can be effective for anxiety and conflict management and increasing job satisfaction [6]. Similarly, in a study conducted by Sun et al. (2020), during the COVID-19 pandemic, among frontline nurses, showed a strong negative association between EI and anxiety, depression, and stress. Nurses with higher EI reported effective management of negative emotions and increased resilience, suggesting that in such way EI can contribute to nurses' mental well-being in highly stressful situations [7].

Organizational culture is also one factor that can impact employees' EI through leadership style, communication and interpersonal relationships at work, and structure. According to Neong et al. (2022), organizational cultures that prioritize knowledge sharing, leadership, and collaborative decision-making tend to nurture emotionally intelligent behavior. On the other hand, in more inflexible, hierarchical organizations, such as those commonly seen in military hospitals, emotional expression may be limited [8]. In line with this assumption, Diamantidou et al. (2024) argue that transformational leadership, which is more likely to be supported in flexible and participatory organizational cultures, fosters EI and a climate of psychological safety, empowerment, and emotional regulation [9]. These findings indicate that hierarchical or control-oriented cultures can avert the expression and development of emotional competencies, while organizations that invest in human capital and encourage adaptive, value-driven leadership foster emotional well-being and relational capacity of their staff. In contrast to strict and hierarchical environments like military hospitals, where emotional expression may be repressed, studies have shown that emotionally intelligent behaviors are more likely to thrive in adaptable and participatory cultures, like those frequently found in public hospitals [8],[10].

In addition, EI can be impacted by occupation. Professions that are associated with emotional labor and patient engagement and require direct interpersonal interaction, such as doctors and nurses, are linked to higher EI [11],[12]. Moreover, gender is a moderating influence in the perception and development of EI. The fact that women are frequently underrated in managerial performance reviews, despite having greater emotional awareness and empathy skills, suggests that gender biases in organizational assessments exist [13],[14]. These results highlight how crucial it is to take gender dynamics, professional hierarchy, and organizational structure into account when assessing or developing interventions to improve EI in healthcare settings.

In the healthcare sector, EI positively affects teamwork, effectiveness, and emotional control. According to Papathanasiou et al. (2021) [15], EI includes skills such as self-awareness, self-control, emotional perception, and sociability. These skills enhance decision-making and the management of professional challenges. We examined the relationship between EI and job boredom among 189 Greek nurses. The findings indicated that higher EI correlates negatively with workplace boredom, suggesting that nurses with high EI cope better with monotonous and repetitive tasks and maintain interest in their work. Additionally, researchers have found a significant relationship between EI and nurses' professional quality of life, particularly in high-pressure environments like prehospital emergency care services [15]. According to Musio et al. (2024) [16], nurses with higher EI reported greater satisfaction with the care they provided and experienced less burnout and post-traumatic stress. Other studies have linked EI to psychological resilience and the prevention of burnout [17]. Asimopoulos et al. (2020) [18] demonstrated that social work students with higher EI exhibited better problem-solving skills and emotional stress management, improving their effectiveness in demanding environments. Healthcare professionals have shown similar results, with EI increasing organizational commitment and decreasing turnover intention [19].

The study by Galanis et al. (2024) [20] confirmed that EI improves nurses' performance in work tasks and teamwork while simultaneously decreasing behaviors detrimental to the work environment and workplace well-being. Research suggests that enhancing EI through educational interventions may improve professional performance and cultivate a positive work environment. In addition, Papathanasiou et al. (2020) [15] emphasized the positive relation between EI and the caring behavior of psychiatric nurses. Higher EI was associated with better caring behaviors and created strong therapeutic relationships. Moreover. Empathy is an essential element of EI, enabling nurses to understand patients' demands more effectively and adapt their approaches accordingly [15].

Despite their different duties, responsibilities, and emotional demands, there are not many comparative studies explicitly looking at the differences in EIEI among healthcare professionals, such as doctors and nurses, although EI has been extensively studied across healthcare professions. While nurses manage patient needs and discomfort, family concerns and team interactions provide ongoing emotional labor, which demands a high level of empathy, emotional perception, and regulation skills [15]. Doctors are often required to make clinical decisions under time constraints, juggling professional judgment with the capacity to maintain emotional control and responsiveness [20]. These distinct professional roles imply that nurses and doctors exhibit EI in distinct ways. In addition to this, the Greek healthcare system is characterized by understaffing, long hours, and high levels of emotional stress, which complicates the expression and development of EI. Although several studies have evaluated EI in each profession separately [15],[20], a direct comparison is required to understand how organizational and profession-specific factors may influence EI. Furthermore, knowing these EI differences between professions may help develop tactics to strengthen interprofessional cooperation, reduce burnout, and improve the quality of care. It is feasible to assume that EI may differ by hospital type (military *vs*. public), job position (managerial *vs*. clinical), and gender due to the influence of professional roles, hierarchical organizational cultures, and gendered emotional norms. According to earlier studies, participatory cultures foster emotional expression while strict, control-oriented workplaces may stifle it [8],[21]. Furthermore, because of the emotional demands of personnel management and decision-making, those in leadership positions or with more job autonomy frequently report higher EI levels [22]. There has also been much discussion about gender differences in EI, with some research showing that women are better at empathy, and others showing that men score higher on self-regulation, perhaps because of social and professional expectations [13],[23]. Our hypotheses about contextual and demographic factors influencing EI were informed by these theoretical stances. Thus, our goal is to evaluate and compare the EIEI levels of Greek doctors and nurses and investigate how these levels relate to their professional and demographic traits.

## 2. Materials and methods

### 2.1. Participants

The study population included nurses and physicians working in the ICUs, and surgical and pathological departments of four Armed Forces and three NHS hospitals in Attica.

### 2.2. Study design

We conducted a cross-sectional study with a 77.8% participation rate (1108 out of 1424). Convenience sampling was performed, as random sampling was impractical due to time constraints. Data was collected from November 2022 to July 2023. The selected nurses and physicians had worked in the departments for at least three months. Questionnaires were distributed to all the nurses and physicians working in the aforementioned departments, and efforts were made to maximize the participation rate. We used G*Power v.3.1.9.2 to calculate the sample size and considered 12 predictors, including demographic and professional characteristics. With an anticipated effect size of 0.02 between the demographic and professional characteristics and the outcome (EI), a statistical power of 99%, and a margin of error of 5%, the sample size was estimated to be 921.

### 2.3. Instrument

We employed the “Wong and Law EI Scale” (WLEIS) to assess EI and questionnaires to gather demographic and professional data. The collected demographic and professional variables included gender, age, marital status, level of education, hospital and department, job title, and years of experience overall and in the hospital and department. Furthermore, data was also gathered regarding the status of permanent employment and working hours.

The WLEIS [1] has been standardised in Greek by Kafetsios and Zampetakis (2008) [4] and Psilopanagioti et al. (2012) [19]. The instrument comprises 16 items assessed on a seven-point Likert scale, ranging from “Strongly Disagree” (1) to “Strongly Agree” (7). According to this scale, EI is described through four dimensions: self-recognition of emotions, recognition of others' emotions, regulation of one's emotions, and application of emotions to enhance performance. The total score and that for each dimension were calculated from the average of responses to the relevant questions. Higher scores indicated a more positive EI assessment, with the minimum and maximum possible scores being 1 and 7, respectively.

### 2.4. Statistical analysis

In the statistical analysis, the categorical variables are presented as absolute (*n*) and relative (%) frequencies, while quantitative variables are presented as mean, standard deviation, median, and interquartile range.

The Kolmogorov-Smirnov test and Q-Q plots were used to test the normal distribution of quantitative variables. All dependent quantitative variables of the study followed a normal distribution. First, we performed bivariate analyses to assess the relationship between demographic and professional characteristics and EI. The independent samples *t*-test (Student's *t*-test) was used to investigate the existence of a relationship between a quantitative variable that followed the normal distribution and a dichotomous variable.

Pearson's correlation coefficient was used to investigate the relationship between two quantitative variables following the normal distribution. The Spearman correlation coefficient was used to investigate the relationship between a quantitative and an ordinal variable (or a quantitative variable that did not follow a normal distribution). Analysis of variance was employed to investigate the relationship between a quantitative and a categorical variable. We then performed a multivariable analysis to eliminate confounding. Dependent variables were quantitative variables that followed a normal distribution, and thus, we performed a multivariable linear regression analysis. A multivariable linear regression with a backward stepwise approach was applied in cases where more than two independent variables were significant at the 0.2 level (*p* < 0.2) in the bivariate analysis. We presented the coefficients b, 95% confidence intervals, and *p*-values. The statistical significance level (*α* value) used in all statistical tests was set at 0.05; thus, a *p*-value < 0.05 was considered statistically significant. Data analysis was performed using IBM SPSS 21.0 (Statistical Package for Social Sciences) for Windows.

### 2.5. Ethics

The National and Kapodistrian University of Athens' Ethics Committee gave its clearance to the study (approval number: 359, June 2021). Additionally, the Ethical Committees of the hospitals that received the surveys granted the required approvals. Following a thorough explanation of the study's design, the medical experts decided on their participation. After that, the medical professionals filled out the survey without divulging any personal information. Because there was no time limit, participants were not hurried and were not forced to make snap decisions. The Declaration of Helsinki's ethical guidelines were strictly followed when conducting the study. As a result, we made every effort to guarantee (a) the healthcare professionals' informed consent, (b) their anonymity, and (c) the confidentiality of the information gathered.

## 3. Results

Of the 1108 medical professionals in the sample (Table 1), 53.4% were employed by military hospitals, and 46.6% were employed by non-military institutions. The proportion of genders was even, with slightly more women than men (52.7% *vs*. 47.3%). On average, the participants were about 38.7 years old. Most respondents were married (59.8%) and worked in clinical settings, primarily as doctors (44.7%) and nurses (48.7%). Regarding education, 19.5% had a master's degree and 28.2% had an MD. Notably, compared to non-military hospitals (58.0%), a larger proportion of employees in military hospitals had permanent posts (80.0%).

### 3.1. EI

The overall EI score and the scores of its four components ranged from 1 to 7, with elevated values signifying enhanced overall EI and increased levels in each dimension, respectively. According to the mean scores, all facets of EI, along with the overall EI score, were evaluated as satisfactory (*mean* score = 5.8 to 6.0, with a maximum score of 7.0). The Cronbach's alpha coefficient for the complete questionnaire was excellent (0.904). Cronbach's alpha coefficient demonstrated acceptable to good reliability across all scales. Consequently, the overall reliability of the questionnaire was considered satisfactory. Table 2 presents descriptive data for the overall score, scale reliability, and the four components of EI.

**Table 1.** The demographic and professional characteristics of the participants.

**Participants Characteristics**	**Military hospitals (*n* = 591, 53.4%)**		**Non-military hospitals (*n* = 517, 46.6%)**		**Total (*n* = 1108)**	

	***Ν***	**%**	***Ν***	**%**	***Ν***	**%**
**Gender**						
Females	298	50.4	286	55.3	584	52.7
Males	293	49.6	231	44.7	524	47.3
**Age**	39.1 (9.7)^a^	39.0 (15.0)^b^	38.2 (10.0)^a^	36.0 (15.5)^b^	38.7 (9.8)^a^	37.5 (16.0)^b^
**Marital Status**						
Singles/divorced/widowed	224	37.9	221	42.7	445	40.2
Married	367	62.1	296	57.3	663	59.8
**Education**						
Nursing Technological Institute Graduate	122	20.6	125	24.2	247	22.3
Nursing University Graduate	148	25.0	90	17.4	238	21.5
MD	147	24.9	165	31.9	312	28.2
MSc	126	21.3	90	17.4	216	19.5
PhD	48	8.1	47	9.1	95	8.6
**Department**						
ICU	111	18.8	148	28.6	259	23.4
Surgical	225	38.1	203	39.3	428	38.6
Medical	255	43.1	166	32.1	421	38.0
**Working Possition**						
Supervisors	45	7.6	28	5.4	73	6.6
Physicians	234	39.6	261	50.5	495	44.7
Nurses	312	52.8	228	44.1	540	48.7
**Working experience in the present hospital**	12.5 (9.8)^a^	11.0 (17.0)^b^	8.7 (8.1)^a^	5.0 (11.0)^b^	10.7 (9.3)^a^	8.0 (15.0)^b^
**Total Working experience**	15.5 (10.1)^a^	15.0 (17.0)^b^	12.4 (9.6)^a^	10.0 (15.0)^b^	14.1 (10.0)^a^	12.0 (16.8)^b^
**Working experience in the department**	6.8 (6.5)^a^	4.0 (8.0)^b^	7.1 (7.1)^a^	4.0 (8.0)^b^	6.9 (6.8)^a^	4.0 (8.0)^b^
**Working status**						
Permanent Position	473	80.0	300	58.0	773	69.8
Non permanent Position	118	20.0	217	42.0	335	30.2
**Shifts**						
Morning shift	240	40.6	90	17.4	330	29.8
Rotated shifts	351	59.4	427	82.6	778	70.2

Note: ^a^ *Mean* (*Standard deviation*), ^b^ *Median* (*Interquartile range*).

### 3.2. Bivariate analysis

The results of the bivariate analysis (Table 3) indicated notable relationships between participant characteristics and EI components. Gender differences were significant for the ‘Use of Emotion’ component (*p* = 0.004), with males showing slightly higher scores. Age showed significant correlations with ‘Self-emotion Appraisal’ (*p* = 0.040) and ‘Use of Emotion’ (*p* = 0.046), suggesting that older participants might perform better in these dimensions. The type of hospital had a substantial impact on all EI components except ‘Regulation of Emotion,’ with participants in NHS hospitals scoring higher overall (*p* < 0.001). Working status was also relevant for ‘Regulation of Emotion’ and ‘Total EI’, where permanent staff had marginally better scores. Shift type also influenced EI, with rotated shifts scoring higher across most components compared to morning shifts (*p* = 0.015). These findings emphasize the role of the workplace and demographic factors in shaping EI among healthcare professionals, suggesting targeted interventions for optimizing EI in clinical settings.

**Table 2.** Descriptive statistics for the emotional intelligence scale.

**Factor**	***Mean***	***Standard deviation***	***Median***	***Min***	***Max***	**Cronbach *a***
**Self-emotion appraisal**	5.8	0.8	6.0	1.8	7.0	0.632
**Other's emotion appraisal**	5.8	0.9	6.0	2.0	7.0	0.721
**Use of emotion**	6.0	0.8	6.3	2.0	7.0	0.732
**Regulation of emotion**	5.9	0.9	6.3	1.3	7.0	0.786
**Total EI**	5.9	0.7	6.1	2.0	7.0	0.904

**Table 3.** Bivariate analysis with emotional intelligence and its dimensions as dependent variables and sociodemographic and professional characteristics as independent variables.

Participants Characteristics	Self-emotion appraisal		Other's emotion appraisal		Use of emotion		Regulation of emotion		Total EI	

	*Mean* (*SD*)	*p*	*Mean* (*SD*)	*p*	*Mean* (*SD*)	*p*	*Mean* (*SD*)	*p*	*Mean* (*SD*)	*p*
Gender^a^		0.488		0.054		0.004		0.202		0.672
Female	5.9 (0.8)		5.9 (0.8)		6.0 (0.8)		5.9 (0.9)		5.9 (0.7)	
Male	5.8 (0.7)		5.8 (0.9)		6.1 (0.7)		6.0 (0.8)		5.9 (0.7)	
Age^b^	0.040^b^	0.188	0.046^b^	0.123	−0.013^b^	0.672	0.038^b^	0.206	0.033^b^	0.279
Marital Status^a^		0.150		0.312		0.903		0.256		0.284
Single/divorced/separate/widowed	5.8 (0.8)		5.8 (0.9)		6.0 (0.8)		5.9 (0.9)		5.9 (0.7)	
Married/cohabited	5.9 (0.7)		5.8 (0.8)		6.0 (0.8)		6.0 (0.9)		5.9 (0.7)	
Education^c^	−0.048^c^	0.109	−0.089^c^	0.003	0.025^c^	0.404	−0.040^c^	0.188	−0.057^c^	0.056
Type of Hospital^a^		<0.001		0.004		<0.001		0.069		<0.001
Military Hospital	5.7 (0.8)		5.8 (0.9)		6.0 (0.8)		5.9 (0.9)		5.8 (0.8)	
NHS Hospital	6.0 (0.7)		5.9 (0.8)		6.1 (0.7)		6.0 (0.8)		6.0 (0.6)	
Department^d^		0.987		0.128		0.767		0.325		0.690
ICU	5.8 (0.7)		5.8 (0.8)		6.1 (0.7)		6.0 (0.7)		5.9 (0.7)	
Surgical	5.8 (0.8)		5.8 (0.9)		6.1 (0.8)		5.9 (0.9)		5.9 (0.7)	
Medical	5.9 (0.8)		5.9 (0.8)		6.0 (0.9)		6.0 (0.9)		5.9 (0.8)	
Working Possition^a^		0.144		0.314		0.870		0.961		0.532
Managers (MD/Nursing). Sector chiefs/head of departments	6.0 (0.7)		5.9 (0.8)		6.0 (0.8)		5.9 (0.8)		6.0 (0.7)	
Attending MDs/ Residents MDs/ Staff nurses	5.8 (0.8)		5.8 (0.9)		6.0 (0.8)		6.0 (0.9)		5.9 (0.7)	
Working experience in the hospital^c^	0.045^c^	0.130	0.088^c^	0.003	0.010^c^	0.748	0.077^c^	0.010	0.074^c^	0.014
Total Working experience ^c^	0.036^c^	0.236	0.045^c^	0.134	−0.015^c^	0.607	0.046^c^	0.127	0.043^c^	0.154
Working experience in the department^c^	0.049^c^	0.104	0.085^c^	0.005	0.027^c^	0.367	0.036^c^	0.237	0.062^c^	0.039
Working status^a^		0.315		0.025		0.922		<0.001		0.035
Permanent Position	5.9(0.8)		5.9 (0.8)		6.0 (0.8)		6.0 (0.8)		5.9 (0.7)	
Not permanent Position	5.8(0.7)		5.7 (0.9)		6.0 (0.8)		5.8 (0.9)		5.8 (0.7)	
Shifts^c^		0.019		0.114		0.015		0.021		0.015
Morning shift	5.8(0.8)		5.8 (0.9)		6.0 (0.8)		5.9 (0.9)		5.8 (0.8)	
Rotated shifts	5.9(0.7)		5.9 (0.8)		6.1 (0.8)		6.0 (0.9)		6.0 (0.7)	

Note: ^a^ *t*-test; ^b^ Pearson r coefficient; ^c^ Spearman rho coefficient; ^d^ *ANOVA*.

### 3.3. Multivariable analysis

Multivariable linear regression analysis (Table 4) revealed several significant relationships between EI dimensions and participant characteristics. In terms of self-emotion appraisal, employees in NHS hospitals scored significantly higher compared to those in military hospitals (*b* = 0.198, *p* < 0.001). Furthermore, lower scores were associated with junior or less senior professional roles (*b* = −0.193, *p* = 0.038). In terms of others' emotional appraisal, a higher level of education was linked to slightly lower scores (*b* = −0.048, *p* = 0.017). Participants in NHS hospitals also scored higher (*b* = 0.212, *p* < 0.001), with those in pathological departments showing improved appraisal skills compared to those in surgical or ICU (*b* = 0.074, *p* = 0.026). In terms of regulation of emotions, male participants scored higher (*b* = 0.151, *p* = 0.001), and participants from NHS hospitals also exhibited higher regulation (b = 0.172, *p* < 0.001). NHS hospital employees showed better utilization of emotions (*b* = 0.119, *p* = 0.029). Permanent staff demonstrated stronger emotional application skills than those in temporary positions (*b* = −0.260, *p* < 0.001), and participants working shifts performed better than those on morning schedules (*b* = 0.136, *p* = 0.020). NHS hospital employees had significantly higher total EI scores compared to military hospital staff (*b* = 0.173, *p* < 0.001), and temporary staff were associated with lower total EI scores (*b* = −0.156, *p* = 0.001).

**Table 4.** Multivariable linear regression with emotional intelligence and its dimensions as dependent variables and sociodemographic and professional characteristics as independent variables.

	**Independent variable**	***b***	**95% confidence interval for *b***	***p*-value**
**Self-emotion appraisal**	NHS Hospital *vs*. Military Hospital	0.198	0.107 to 0.288	**<0.001**
	Clinical Supervisor. Clinical Physician (Specialist). Clinical Physician (Resident). Nurse *vs*. Director of Medical Services. Nursing Services. Clinic. Sector Manager. Department Head	−0.193	−0.375 to −0.011	**0.038**
**Other's emotion appraisal**	Educational level	−0.048	−0.088 to −0.009	**0.017**
	NHS Hospital *vs*. Military Hospital	0.212	0.107 to 0.316	**<0.001**
	Medical Department *vs*. other	0.074	0.009 to 0.140	**0.026**
**Regulation of emotion**	Male *vs*. female	0.151	0.058 to 0.244	**0.001**
	NHS Hospital *vs*. Military Hospital	0.172	0.076 to 0.268	**<0.001**
**Use of emotion**	NHS Hospital *vs*. Military Hospital	0.119	0.012 to 0.225	**0.029**
	Permanent position *vs*. non-permanent	−0.260	−0.373 to −0.147	**<0.001**
	Night shifts *vs*. morning shifts	0.136	0.022 to 0.249	**0.020**
**Total EI**	NHS Hospital *vs*. Military Hospital	0.173	0.086 to 0.260	**<0.001**
	Permanent position *vs*. non-permanent	−0.156	−0.248 to −0.063	**0.001**

## 4. Discussion

We investigated the EI of healthcare professionals in ICUs and three surgical and medical departments across four military and three NHS hospitals in Attica. A cross-sectional study was performed with a participation rate of 77.8%, employing convenience sampling. The collection of data lasted from November 2022 to July 2023.

In this study, conducted for the first time in Greece, we compared the EI of healthcare workers in the NHS and Armed Forces hospitals. The data revealed that the personnel in NHS hospitals possessed higher EI overall and across all individual characteristics. NHS personnel rated their EI more positively than their counterparts in Armed Forces hospitals. Furthermore, regarding the self-awareness aspect of EI, clinical supervisors, senior and junior physicians, and nurses rendered lower assessments than directors of medical, nursing, or clinical services, department heads, or section supervisors. In addition to EI scores, there were notable differences in the staffing levels between NHS and military hospitals. The percentage of female employees, temporary workers, and staff on rotational shifts was greater in NHS hospitals. When evaluating the results, it is important to consider these structural variations, which could help to explain some of the variances in EI.

EI is frequently regarded as a feminine characteristic [3],[23]. Research indicates contradictory results concerning EI differences between genders [17]. This study revealed that men have a greater overall EI than women, challenging the belief that EI is a benefit of female leadership. However, other research has not found notable gender differences regarding overall EI [24]. Mayer & Geher (1996) [25] suggested that women achieve better scores in EI assessments. This particularly intriguing finding contrasts with several studies that report a female advantage in emotional awareness and empathy. Our results, however, are consistent with research by Papathanasiou et al. (2021) [17], who found that men had higher EI ratings. These disparities could be explained by gender norms, cultural influences, or expectations of professional roles in Greek healthcare environments. It is possible that male professionals in male-dominated settings, such as military hospitals, have improved their emotional regulation as a coping mechanism, particularly in reaction to emotionally taxing and hierarchical work arrangements. Alternatively, self-report responses might have been impacted by gendered judgments of emotional competence or social desirability bias [10],[13],[14],[23]. In contrast, Frixou (2019) [26] indicated that women of the Cypriot population surpassed males in EI ratings. Katinić et al. (2022) reported analogous findings, indicating statistically significant emotional advantages for women compared to men. Additional international literature indicates that women often have a marginal advantage in overall EI [27]. Nevertheless, studies conducted across several nations revealed that men demonstrated higher EI scores compared to women [22],[23],[28]. Jurado et al. (2019) highlighted the importance of administrators' EI in the efficiency of healthcare services, emphasizing the lack of relevant research in international literature. Moreover, they suggested that EI should be included as an important skill in training curricula to improve the healthcare professionals' ability to manage emotions in patient care and relationships with patients, families, and colleagues [22]. Therefore, to better understand the lived experiences that underlie these findings, more studies should examine the relationship between organizational setting, gender, and professional hierarchy, maybe using qualitative data.

NHS hospital employees exhibited higher EI across all dimensions, which highlights the significant influence of organizational culture on EI. In NHS hospitals, the emphasis on teamwork and communication fosters an environment conducive to EI development and expression. This agrees with McGuier et al.'s (2024) [29] findings linking high levels of EI to adaptive team functioning and open communication correlate with improved healthcare outcomes. Conversely, the rigid hierarchy and structured organization in military hospitals may limit EI development by poor interpersonal interactions and fewer collaborative decision-making opportunities [29]. Studies in military healthcare teams underline the challenges posed by strictly defined roles and power dynamics to cohesion and team performance. Furthermore, Ballangrud et al. (2017) [30] underline the importance of collaborative teamwork and mutual respect in effective care delivery, factors that may be lacking in more hierarchical systems. Specific tailored interventions for military hospitals could integrate strategies to foster EI by reducing hierarchical barriers and promoting collaborative training, such as simulations, to improve team cohesion and adaptability. Such initiatives could bridge the EI gap between NHS and military hospital employees, enhancing individual and organizational outcomes [30].

EI in healthcare settings is significantly shaped by leadership style and organizational structure. Over the years, transformational leadership has been linked to the enhancement of emotional awareness and regulation among healthcare workers. Transformational leadership is typified by motivation, personalized attention, and inspirational communication [9]. Public sector hospitals, such as those in the NHS, often foster participatory decision-making and leadership models that promote psychological safety and emotional development [8],[20]. On the other hand, the strict and hierarchical management style that is commonly found in military hospitals seems to affect social and interpersonal relationships and emotional expression, two essential elements of EI. Therefore, leadership development, emphasizing empathy, EI, and inclusive communication, may be a tool for enhancing EI in organizational contexts. These results are in line with research that indicates EI leadership fosters a workforce that is more resilient, involved, and cooperative [10],[11].

### 4.1. Implications for practice

Our findings can have significant implications for healthcare practice and policy, especially in settings like military hospitals that are emotionally taxing and hierarchically organized. Since they may increase job satisfaction, lower burnout, and promote greater interprofessional collaboration, tailored training programs targeted at improving EI should be integrated into doctors' and nurses' professional development. In addition, this study emphasizes the significance of leadership models in clinical contexts and advocates for leadership styles that foster emotional engagement, psychological safety, and participatory decision-making to develop emotionally intelligent organizational cultures.

### 4.2. Limitations

This study has some limitations. First, the convenience sampling procedure does not enable a representative sample of healthcare workers and generalization of the results. Furthermore, the cross-sectional design does not reveal any causal links between EI and the demographic or professional characteristics of the sample. Finally, the quantitative nature of this research limits the capacity to understand the underlying context, individual interpretations, and deeper implications of the participants' responses. In future studies, researchers should use a mixed-methods approach to overcome these constraints, integrating qualitative methods like focus groups and individual interviews with quantitative measures to provide more thorough knowledge of EI in healthcare contexts.

## 5. Conclusions

The study highlighted the significance of EI among healthcare professionals in Greece, identifying substantial differences between NHS and Military hospitals. NHS employees demonstrated higher overall EI and scores across all dimensions, underscoring the positive impact of a communication- and collaboration-friendly environment. In contrast, the stricter hierarchy of military hospitals appears to restrict EI development. The analysis also revealed relationships between EI and demographic or professional characteristics. Men showed higher performance in emotional regulation and overall EI, in contrast to the literature that suggests women report higher EI compared to men. Employees in permanent positions or with more professional experience demonstrated higher EI, indicating the value of stability and experience in developing EI. Finally, this study contributes to the literature by demonstrating that EI is a vital factor that improves professional quality of life and quality of healthcare. The findings highlight the need to foster EI through tailored training programs that address the unique characteristics of each work environment. For military hospitals, educational initiatives promoting collaboration and empathy are recommended. Further exploration of gender differences, and the relationship between EI and professional stability is necessary to design targeted strategies that reduce occupational stress and fatigue while enhancing healthcare quality. Overall, EI can contribute to creating a supportive work environment that fosters professional development and delivery of high-quality care.

## Use of AI tools declaration

The authors declare they have not used Artificial Intelligence (AI) tools in the creation of this article.
